# Integrated Extraction of Tea Tree Oil and Polysaccharides From *Melaleuca alternifolia* Using Three‐Phase Partitioning: Process Optimization, Techno‐Economic Analysis, and Sustainability Evaluation

**DOI:** 10.1002/cbdv.71748

**Published:** 2026-09-25

**Authors:** Huan Liao, Simin Wang, Ying Wu, Zhihong Chen, Wentao Chen, Leiwen Xiang, Yangmin Wen, Youcai Zhou

**Affiliations:** ^1^ School of Food and Biological Engineering Fujian Polytechnic Normal University Fuqing China; ^2^ Quanzhou Medical College Quanzhou China

**Keywords:** polysaccharides, response surface methodology, sustainable extraction, tea tree oil, three‐phase partitioning

## Abstract

Conventional tea tree oil extraction approaches such as steam distillation are limited by excessive water and energy input, alongside the production of polysaccharide‐rich waste residues. This study systematically explored the application of three‐phase partition (TPP) method in *Melaleuca alternifolia* for integrated extraction of tea tree oil (TTO) and polysaccharides, addressing challenges in conventional methods. Using ammonium sulfate ((NH_4_)_2_SO_4_) and *tert*‐butanol (*t*‐butanol), the TPP process operated under mild conditions (60°C). Through Box–Behnken Design optimization, TPP methods achieved 1.15% TTO and 5.57% polysaccharide yields rich in neutral pentoses. GC‐MS analysis identified 32 volatile compounds in TPP‐derived TTO, among which terpinen‐4‐ol (33.19%) served as the dominant bioactive constituent and satisfied the 30%–48% threshold specified in GB/T 26514‐2011, while multiple minor terpene components fell outside the standard concentration ranges. A techno‐economic evaluation revealed that the integrated process incurred a total direct material and electricity cost of 176.6 RMB for extracting 11.5 g of TTO and 55.7 g of polysaccharides, with *t*‐butanol expenses constituting the majority (65.6%) of costs. Overall, this study is the first to establish a TPP process for simultaneous extraction of TTO and polysaccharides from *M. alternifolia*, offering a green, economically viable, and sustainable approach for industrial‐scale bioactive compound recovery.

## Graphical abstract

This mild three‐phase partitioning method simultaneously recovers tea tree oil and heteropolysaccharides from *Melaleuca alternifolia*. Optimized parameters are validated at a 30 L pilot scale; the key bioactive terpinen‐4‐ol in oil meets national standards, and techno‐economic analysis reveals its promising green industrial potential.

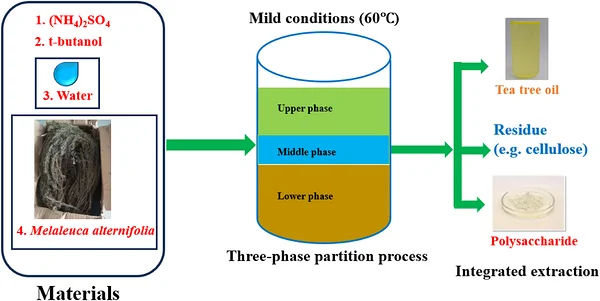

## 1 Introduction

*Melaleuca alternifolia* (tea tree), native to Australia, has gained global recognition for its therapeutic properties, primarily attributed to tea tree oil (TTO), a complex mixture of volatile organic compounds (VOCs) dominated by terpinen‐4‐ol (30%–40%), α‐terpinene, and 1,8‐cineole [1, 2]. Since its introduction to China in the 1980s, *M. alternifolia* has been cultivated across Guangdong, Fujian, and Yunnan provinces, with TTO widely used in pharmaceuticals, cosmetics, and food additives due to its broad‐spectrum antimicrobial and anti‐inflammatory activities [3]. For instance, TTO has broad‐spectrum activity against bacteria and fungi, and TTO products have been evaluated clinically for a range of conditions and infections, including the treatment of chronic infected wounds and for the elimination of Methicillin‐resistant *Staphylococcus aureus* [4]. Furthermore, the likelihood of bacteria developing resistance to TTO is extremely low, attributed largely to the cell membrane being a major site of action [5].

Despite the proven therapeutic application of TTO, the efficiency and sustainability of TTO extraction remain critical challenges for industrial‐scale production. Nowadays, the dominant method of steam distillation for TTO extraction requires prolonged heating and substantial water consumption [6]. Alternative techniques such as supercritical CO_2_ extraction require costly specialized equipment and ultrahigh operating pressures (>30 MPa), which raises overall production costs and restricts large‐scale industrial promotion [7]. On the other hand, it is worth noting that *M. alternifolia* biomass contains abundant plant polysaccharides in addition to TTO [8]. However, conventional extraction methods (e.g., steam distillation) focus solely on TTO recovery, resulting in the discarding of residual polysaccharide‐rich biomass as waste. This practice undermines resource efficiency and runs counter to the principles of circular bioeconomy.

Three‐phase partitioning (TPP) is a mild liquid‐liquid separation technique proven effective for co‐extracting lipophilic oils and hydrophilic polysaccharides from numerous plant matrices [9]. By adding ammonium sulfate ((NH_4_)_2_SO_4_) and *tert*‐butanol (*t*‐butanol) to an aqueous plant suspension, TPP forms three distinct phases [10]: upper *t*‐butanol‐rich phase that enriches hydrophobic compounds (e.g., lipids); middle solid phase that contains proteins and macromolecules; and lower (NH_4_)_2_SO_4_‐rich phase that precipitates polysaccharides and other hydrophilic substances. TPP method offers several advantages [11]: (1) mild operational conditions that room‐temperature extraction preserves thermolabile components; (2) simultaneous product recovery that extracts both lipids (upper phase) and polysaccharides (lower phase) in a single step; (3) solvent recyclability that *t*‐butanol can be recovered and reused for five cycles, reducing costs by ∼80%; (4) environmental sustainability that *t*‐butanol has low volatility (bp: 84°C) and minimal toxicity compared to conventional solvents like hexane.

Despite the numerous advantages of the TPP method, its application in *M. alternifoli*a processing remains underexplored. Prior studies have optimized ultrasonic or enzymatic extraction of *M. alternifolia* polysaccharides, while no systematic research has evaluated TPP for the co‐extraction of TTO and polysaccharides [8]. In addition, critical parameters including (NH_4_)_2_SO_4_ concentration, *t*‐butanol ‐to‐water ratio, and extraction time strongly affect extraction yield, yet their interactive effects are still unclear. Accordingly, this study investigates whether a TPP‐based integrated process enables simultaneous high‐yield recovery of TTO and polysaccharides from *M. alternifolia* under mild conditions, while preserving oil quality and delivering economically viable scale‐up performance. Upon these, this study aims to: (1) screen key yield‐influencing factors via Plackett–Burman Design; (2) optimize TPP parameters using Box–Behnken Design coupled with response surface methodology to maximize co‐yields of TTO and polysaccharides; (3) scale up the process to a 30 L reactor to conduct techno‐economic assessment. By addressing these gaps, a green, integrated extraction system capable of producing high‐value bioactives from *M. alternifolia* biomass while minimizing environmental impact and operational costs was proposed.

## 2 Results and Discussion

### 2.1 Screening of Significant Factors Affecting the TTO Extraction Efficacy Using Plackett–Burman Design

The statistical analysis of how the seven factors examined in this study influenced TTO by the TPP method was conducted using the Plackett–Burman Design. The experimental data underwent statistical analysis through a t‐test for ANOVA, with the findings detailed in Table 1. The R^2^ value of this model was 0.9236 indicating 92.36% variability in TTO extraction could be calculated and the experimental data can be explained by this model. *P*‐values (representing the probability > t) served as indicators to assess the significance of each parameter. Consequently, *P*‐values below 0.05 signified that the factors had a significant impact on the yield of TTO (Y).

**TABLE 1:** Analyses of variance for Plackett–Burman design.

Factors	T‐test	Prob > |*t*|	Significance
(NH_4_)_2_SO_4_, *t*‐butanol	7.28, 5.94	0.002, 0.007	1, 2
Time	4.38	0.032	3
pH	3.85	0.366	4
Temperature	0.99	0.540	6
Solid‐liquid ratio	0.66	0.818	7
Stirring speed	0.24	0.420	5
Error item	0.96	0.395	—

*Note*: R^2^ = 0.9236.

Table 1 showed that the T‐values of six factors were all greater than 0, indicating that these factors have a positive influence on the extraction efficiency of TTO from *M. alternifolia* using the TPP method. However, as shown in Table 1, the factors, including the ratio of raw material to water (g/mL), pH, extraction temperature (°C), and stirring speed (rpm), have no significant effect on TTO extraction. On the other hand, the factors (NH_4_)_2_SO_4_ to water (g/mL), the ratio of *t*‐butanol to water (v/v), and extraction time (h) were found to have significant effects on the yield of TTO extraction and were therefore chosen for the next optimization step. Additionally, since preliminary analysis indicated that the extraction parameters, including the ratio of raw material to water, pH, extraction temperature, and stirring speed that exhibited no significant influence on TTO yield (*p *> 0.05), these parameters were optimized for cost‐efficiency in subsequent experiments. To stabilize the extraction environment and reduce unnecessary energy and raw material costs, the raw material‐to‐water ratio was fixed at 1:10 (w/v), the extraction temperature at 60°C to protect the thermosensitive components (e.g., terpenes) in TTO from degradation, the pH remained at natural conditions, and the stirring speed was set to 50 rpm.

## 3 The Optimal Conditions for TTO Extraction From *M. alternifolia*

### 3.1 Optimization of TTO Extraction Process Using Box–Behnken Design

Based on the identification of key factors through Plackett–Burman design, the TTO extraction process was optimized using Box‐Behnken experimental design. Table S1 presents the three levels of (NH_4_)_2_SO_4_ to water, the ratio of *t*‐butanol to water (v/v), and extraction time, as well as the extraction efficiency of TTO under various experimental conditions. Then, we performed the analysis of variance of the data in Table S1, and the results were shown in Table 2. Table 2 showed that the experimental model difference was highly significant (*p *< 0.01). Meanwhile, there was no significant difference in the lack‐of‐fit term of the model (*p *= 0.1164 > 0.05), indicating that the selected model demonstrated good fit and adequately captured the experimental results. Moreover, in this model, the coefficient of determination (*R* ^2^) was 0.918, and the adjusted *R* ^2^ (Adj *R* ^2^) was 0.8126. The high consistency between the two values proves this model is applicable to optimize the TPP extraction of tea tree oil from *M. alternifolia*. In this optimized model, only the linear term X_1_ ((NH_4_)_2_SO_4_ to water ratio) significantly affected the response variable (*p *< 0.05). This aligns with the findings from the Plackett–Burman design, which showed that the (NH_4_)_2_SO_4_ concentration had a statistically significant impact on TTO yield (*p *< 0.05). Moreover, in the Box–Behnken model, the linear term coefficient of the (NH_4_)_2_SO_4_ concentration was the largest (0.11), suggesting a positive correlation and the highest sensitivity of its influence on the yield. While, none of the other linear terms reached extreme significance (*p* > 0.01). For the interaction terms, none achieved statistical significance (*p *> 0.05). In contrast, all quadratic terms demonstrated extreme significance (*p *< 0.01). The quadratic polynomial equation was derived through stepwise regression analysis as Equation (1):

(1) $$\begin{array}{ccc} Y & = & +2.05+0.11\times X_{1}+0.047\times X_{2}+0.015\times X_{3}-0.015\times X_{1}\times X_{2}\\ & & +\;0.040\times X_{1}\times X_{3}-0.005\times X_{2}\times X_{3}-0.15\times {X_{1}}^{2}-0.14\times {X_{2}}^{2}\\ & & -\;0.12\times {X_{3}}^{2} \end{array}$$

**TABLE 2:** The regression equation of coefficient estimates.

Source	Sum of squares	Degree of freedom	Mean square	F Value	*p*‐value Prob > F
Modle	0.39	9	0.043	8.71	0.0047
X_1_	0.097	1	0.097	19.51	0.0031
X_2_	0.018	1	0.018	3.64	0.0981
X_3_	0.003	1	0.003	0.36	0.5659
X_1_X_2_	0.008	1	0.008	0.18	0.6830
X_1_X_3_	0.006	1	0.006	1.29	0.2934
X_2_X_3_	0.001	1	0.001	0.020	0.8911
X_1_^2^	0.097	1	0.097	19.48	0.0031
X_2_^2^	0.078	1	0.078	15.81	0.0053
X_3_^2^	0.062	1	0.062	12.53	0.0095
Residual	0.035	7	0.002		
Lack of fit	0.026	3	0.001	3.77	0.1164
Pure error	0.0001	4	0.0001		
Cor total	0.043	16			

*Note*: R2, 0.918; Adj.*R* ^2^, 0.8126; prob > F: 0.1164.

**FIGURE 1:**
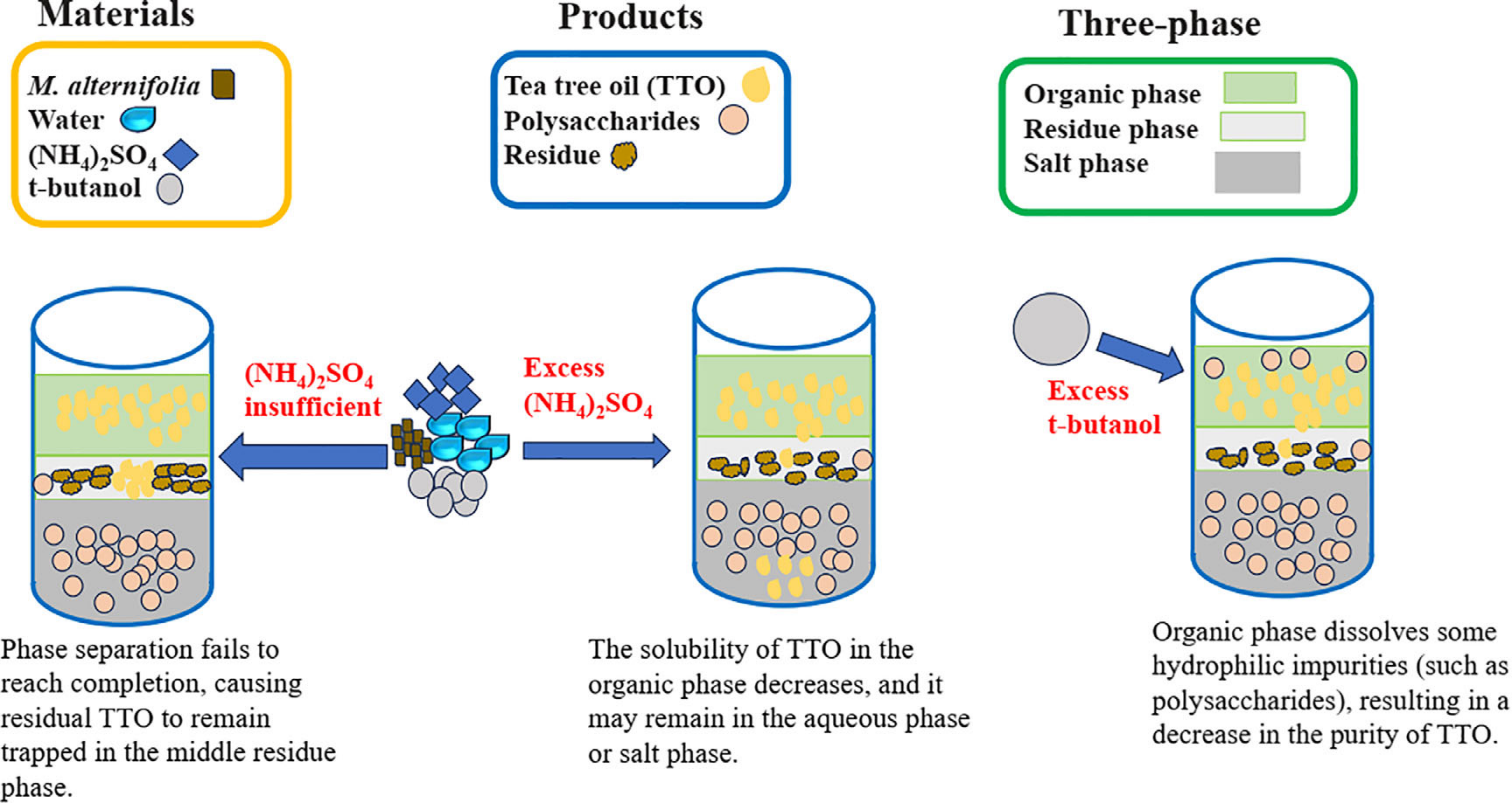
Schematic diagram of the mechanisms of TPP method mediated with (NH_4_)_2_SO_4_ and *t*‐butanol to extract TTO.

The (NH_4_)_2_SO_4_ plays a crucial role in the TPP process by modulating target‐compound partitioning between aqueous and organic phases. At insufficient (NH_4_)_2_SO_4_ concentrations, phase separation cannot be completed [12], causing residual oil to remain trapped in the middle residue phase. A specific quantity of (NH_4_)_2_SO_4_ elevates the interfacial tension between the two solvents, facilitating the separation of *t*‐butanol from the aqueous phase and, consequently, from the oil [13]. In the present work, (NH_4_)_2_SO_4_ exerts dual regulatory effects on the two target products from *M. alternifolia* (Figure 1). As shown in Figure 1, insufficient (NH_4_)_2_SO_4_ fails to establish an effective salting‑out environment, resulting in homogeneous dispersion of TTO and polysaccharides within the mixed system and incomplete phase separation. By contrast, excess (NH_4_)_2_SO_4_ depletes free water, lowers the solvation capacity of *t‐*butanol toward medium‐polarity terpenoids, and suppresses TTO accumulation in the organic phase [14]. With a linear coefficient of 0.11 (Equation (1)), the highest among all variables, (NH_4_)_2_SO_4_ concentration (X_1_) dominates salting‐out‐driven TTO partitioning. Meanwhile, the highly significant negative quadratic term X_1_^2^ (−0.15, *p *< 0.01) indicates yield decline under oversalted conditions. Below 0.20 g/mL(NH_4_)_2_SO_4_, inadequate salting‐out sequesters medium‐polar terpenoids in intermediate residues, lowering the predicted TTO yield below 1.10%. When salt concentration exceeds 0.30 g/mL, free‐water depletion raises organic‐phase polarity, weakens terpenoid solvation, and drives part of TTO back into the salt phase, reducing recoverable oil below 1.15%. These observations are consistent with earlier TPP findings reported by Qiu et al. [15], where salt‐induced dehydration increases organic‐phase polarity and hinders dissolution of nonpolar terpenes, retaining such components in aqueous or salt‐rich layers [16].

As an organic‐phase solvent, *t*‐butanol exerts its function through its solubilizing capacity and phase volume ratio [17]. When this ratio exceeds a certain threshold, increasing the solvent volume will not significantly enhance the yield; instead, it leads to a rise in subsequent recovery costs [18]. Meanwhile, an excessively high proportion of *t*‐butanol may dissolve some hydrophilic impurities (such as polysaccharides) [19]. In the Box–Behnken model, the linear term coefficient (0.047) of the *t*‐butanol proportion is smaller than that of (NH_4_)_2_SO_4_, and the interaction term (X_1_X_2_) between it and (NH_4_)_2_SO_4_ has an insignificant impact on the yield (*p* > 0.05), indicating that its effect is more independent and of lower intensity. The role of extraction time is constrained by kinetic limitations and equilibrium conditions. Phase separation in the TPP system is typically completed within 30–90 min [20], and extending the extraction time will not significantly increase the amount of TTO transferred from the plant material to the organic phase, as the partition equilibrium has been reached. In the Box–Behnken model, the linear term coefficient (0.015) of extraction time is the smallest, and its quadratic term coefficient is negative (−0.12), indicating that excessive prolongation of the extraction time may reduce the yield.

The core of TPP for extracting TTO lies in the phase distribution driven by salting‐out. (NH_4_)_2_SO_4_ plays a dominant role in directing the transfer of target components by altering the solution environment, while *t*‐butanol merely serves as the receiving phase to optimize the dissolution efficiency. Extraction time, categorized as a process parameter, quickly reaches a point of diminishing returns in terms of its impact once phase separation is completed.

### 3.2 Response Surface Analysis of the Effects of Factor Interactions on TTO Yield

Design‐Expert 8.0.6 software was employed to generate response surface plots based on the experimental results. These plots enable a visual analysis of the impact of individual factors as well as the interactions between factors on the response value. The 3D response surface plots and contour plots can visually reveal the strength of interactions among various factors. Specifically, the steeper the slope of the response surface and the more elliptical the contour plot, the more significant the interaction between the factors. Conversely, this indicates that there is no significant difference in the interaction between the two factors.

**FIGURE 2:**
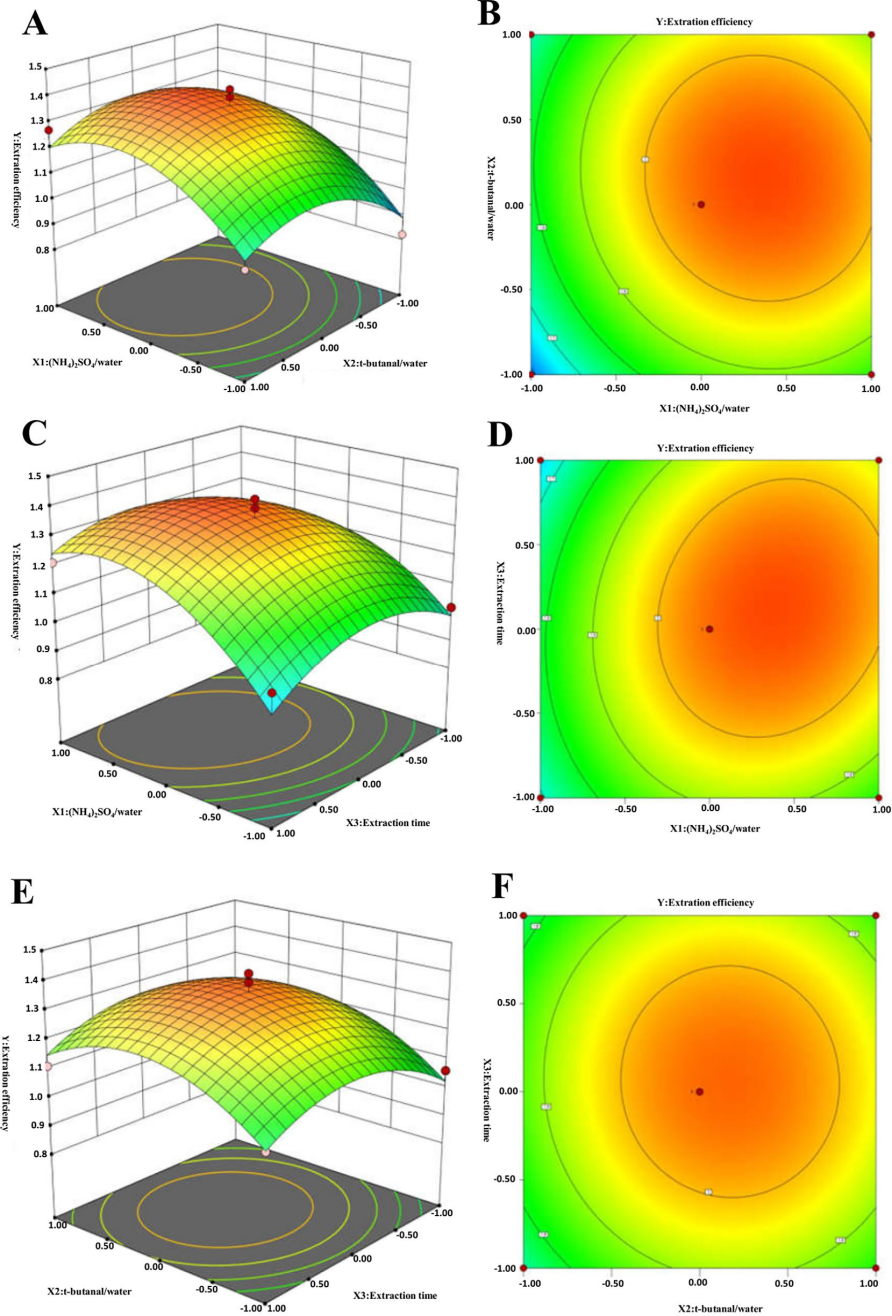
Response surface plots and corresponding contour plots showing the interactive effects of two variables on TTO yield. (A) Interaction between (NH_4_)_2_SO_4_ concentration and *t*‐butanol‐to‐liquid ratio; (B) interaction between (NH_4_)_2_SO_4_ concentration and solid‐to‐liquid ratio; (C) interaction between t‐butanol‐to‐liquid ratio and solid‐to‐liquid ratio.

Figure 2 showed the effects of the remaining two variables on the TTO yield when any one of the three factors ((NH_4_)_2_SO_4_/water ratio, *t*‐butanolwater ratio, and extraction time) was held at the 0 level. The 3D response surface plots all exhibit a parabolic shape with a highest point on the surface, indicating the presence of a maximum value. Figure 2A,B demonstrated that when X_1_ or X_2_ changes independently, the yield of TTO displayed a parabolic variation pattern. When these two factors interact, the slope of the response surface increased significantly, and the densely packed contour lines (with an elliptical shape) suggested a significant interaction between them. As shown in Figure 2A, at a low (NH_4_)_2_SO_4_ concentration, even when the proportion of t‐butanol was increased, the improvement in TTO yield was limited. A possible reason is that insufficient salting‐out results in the retention of some TTO in the aqueous phase. Under a high (NH_4_)_2_SO_4_ concentration, moderately increasing the proportion of *t*‐butanol can significantly enhance the TTO yield. However, an excessive amount of *t*‐butanol will dilute the concentration of TTO in the organic phase (resulting in a decrease in the partition coefficient Kd).

As shown in Figure 2C,D, the response surface curve for X_1_ and X_3_ is also relatively steep, suggesting that the interaction between X_1_ and X_3_ also has a certain impact on the TTO yield. At a high concentration of (NH_4_)_2_SO_4_, the salting‐out process takes place swiftly, leading to the rapid transfer of TTO into the organic phase. Nevertheless, an adequate duration is necessary to overcome the interfacial resistance. When the time was inadequate (for instance, less than 60 min), the yield of TTO was constrained. Conversely, as the process was prolonged excessively (beyond 120 min), thermosensitive components, exemplified by 1,8‐cineole, were prone to degradation [21]. Conversely, when the (NH_4_)_2_SO_4_ concentration was low, even extending the extraction time cannot compensate for the insufficient salting‐out effect, resulting in a persistently low TTO yield. Moreover, the interaction effect under this condition was not statistically significant (Figure 2C). The *p*‐value associated with the interaction term X_1_X_3_ was determined to be 0.293, which is greater than 0.05. This result implied that the interaction between X_1_ and X_3_ does not hold statistical significance. In contrast, the *p*‐values for the quadratic terms X_1_^2^ and X_3_^2^ were both below 0.01. This finding indicated that each individual factor exerts a more pronounced nonlinear influence on the TTO yield (Table 2).

The response surface of X_2_ and X_3_ was relatively gentle, with loosely spaced contour lines, indicating that the interaction between X_2_ and X_3_ has a relatively minor impact on the TTO yield (Figure 2E,F). When the dominance of the *t*‐butanol proportion, the dissolution equilibrium of TTO in the organic phase is generally attained within 30–90 min. Prolonging the extraction time will not lead to a substantial rise in the dissolved quantity; instead, it may induce thermal degradation. Regarding the time threshold effect, once the extraction time surpasses 90 min, the growth rate of the yield declines and becomes independent of the *t*‐butanol proportion. The *p*‐value for the interaction term X_2_X_3_ was 0.891 ( > 0.05), indicating no statistical significance. This further confirmed that the effect of this interaction can be disregarded (Table 2). Overall, the order of influence magnitude of the interactions on the TTO yield is as follows: X_1_‐X_2_ > X_1_‐X_3_ > X_2_‐X_3_.

In summary, the three variables affect TTO yield in the order X_1_ > X_2_ > X_3_, supported by the statistical results in Table 2. The X_1_ ((NH_4_)_2_SO_4_ concentration) showed the largest linear sum of squares (0.097) and regression coefficient (0.11), representing its dominant role in salting‐out partitioning. X_2_ (*t*‐butanol ratio) had a weaker linear contribution (coefficient = 0.047, *p *= 0.0981), while X_3_ (extraction time) exerted the slightest linear influence (coefficient = 0.015, *p *= 0.5659). The quadratic terms also follow the same descending magnitude: X_1_^2^ (−0.15) > X_2_^2^ (−0.14) > X_3_^2^ (−0.12), verifying (NH_4_)_2_SO_4_ concentration as the most vital parameter for TPP extraction.

### 3.3 The Prediction and Verification for the Optimal Conditions

**FIGURE 3:**
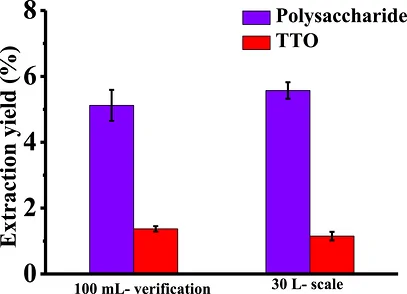
The optimized process for polysaccharide and TTO extraction at 100 mL and 30 L—scale conditions (*n* = 3).

The optimal process for extracting TTO from *M. alternifolia* using the TPP method, as predicted by the regression model, was as follows: (NH_4_)_2_SO_4_ to water ratio of 0.23 g/mL, *t*‐butanol to water ratio of 1.65 mL/mL, and an extraction time of 97.2 min. Under these conditions, the maximum theoretical yield of TTO was 1.41%. Considering the practical constraints and operational convenience of the experiment, the optimal conditions were adjusted to: a (NH_4_)_2_SO_4_ to water ratio of 0.20 g/mL, a *t*‐butanol to water ratio of 1.60 mL/mL, and an extraction time of 90 min. Under these conditions, three parallel experiments were conducted for verification, yielding an average TTO recovery rate of 1.37% (Figure 3). The error between this experimental value and the theoretically predicted value of 1.41% was only 0.04%, confirming the validity of the model. Meanwhile, the lower salt‐rich phase was collected, concentrated, and purified to obtain polysaccharides with a yield of 5.12%. Xiao et al. employed Response Surface Methodology to optimize the ultrasonic extraction of polysaccharides from *M. alternifolia*, achieving a maximum yield of 6.29% [8]. Although the polysaccharide yield in this study was lower than that reported by Xiao et al., our process simultaneously extracted TTO from *M. alternifolia* biomass. Based on the TPP extraction method, simultaneous extraction of polysaccharides and TTO from *M. alternifolia* was achieved for the first time.

### 3.4 Simultaneous Extraction of TTO and Polysaccharides From *M. alternifolia* Using a 30 L Reactor

It has been reported that a 30 L reactor can be used for stability evaluation of extraction processes at the preliminary pilot‐scale level [22]. The process parameters for TPP extraction of TTO from *M. alternifolia*, obtained after response surface optimization, were scaled up to a 30 L scale for simultaneous extraction of TTO and polysaccharides. As shown in Figure 3, in the 30 L scale system, the extraction yields of TTO and polysaccharides from *M. alternifolia* using the TPP extraction method were 1.15% and 5.57%, respectively. It was clear that the extraction process in the larger reactor maintained comparable efficiency for polysaccharides and TTO yield without obvious variations (Figure 3). This indicated the scalability of the extraction process for obtaining both TTO and polysaccharides from *M. alternifolia* using TPP method.

Table S2 listed the compositional profile of extracted TTO, with 32 volatile compounds identified in total. The dominant constituents were terpinen‐4‐ol (33.19%), *β*‐pinene (30.70%), α‐terpinene (14.96%), 1,8‐cineole (6.17%), α‐terpineol (5.23%), and γ‐terpinene (3.73%). A previous study indicated that qualified TTO requires  ≥ 30% terpinen‐4‐ol and  ≤ 15% 1,8‐cineole; both constituents confer strong antimicrobial and anti‐bioactivity [23]. Table 3 further compared TPP‐extracted TTO volatiles against GB/T 26514‐2011. Terpinen‐4‐ol content in TPP‐TTO (33.19%) complied with the standard 30%–48% range, lower than the 40.09% measured for steam‐distilled TTO. Steam distillation afforded a more abundant volatile spectrum, including sabinene and limonene, with most monoterpenes within standard concentration ranges. These two compounds were absent in TPP extracts, while *p*‐cymene, *γ*‐terpinene, and terpinolene were below the standard lower limits. Such differences originate from selective partitioning in the three‐phase extraction system rather than thermal degradation. Taken together, only the core marker terpinen‐4‐ol of TPP‐obtained TTO meets the national standard requirements, while several minor terpene components deviate from the standard ranges. Subsequent research will carry out systematic functional assays, including antioxidant and antibacterial tests, to clarify the biological activity characteristics of TPP‐derived TTO.

**TABLE 3:** Volatile composition comparison of TTO extracted via TPP and steam distillation against GB/T 26514‐2011 national standard.

No.	Components	Yield by TPP (%)	Yield by steam distillation (%) [24]	GB/T 26514‐2011 (%)
1	*α*—Pinene	1.09	5.86	1–6
2	Sabinene	—	0.20	Trace–3.5
3	*α*‐Terpinene	5.23	11.34	5–13
4	Limonene	—	1.36	0.5–1.5
5	*p*‐Cymene	0.02	1.20	0.5–8
6	1,8‐cineole	6.17	1.83	Trace‐15
7	*γ*‐Terpinene	3.73	21.85	10–28
8	Terpinolene	0.33	3.24	1.5‐5
9	Terpinen‐4‐ol	33.19	40.09	30–48
10	*α*‐Terpineol	5.23	6.91	1.5–8

The monosaccharide composition of TPP‐extracted *M. alternifolia* polysaccharides was quantitatively analyzed via ion chromatography (IC), and the relative molar proportions of each component are presented in Table 4.

**TABLE 4:** Monosaccharide composition and molar proportion of polysaccharides extracted from *M. alternifolia* via the TPP method.

NO.	Retention time (min)	Components	Proportion (%)
1	3.44	Fucose	13.30
2	5.81	Rhamnose	10.95
3	6.32	Arabinose	14.20
4	7.75	Galactose	13.46
5	8.50	Glucose	13.96
6	9.63	Xylose	16.54
7	11.10	Fructose	6.73
8	25.26	Galacturonic acid	3.90
9	26.69	Glucuronic acid	6.97
Total			100

Nine monosaccharide constituents were identified in total, consisting of seven neutral monosaccharides and two uronic acids. Xylose was the most abundant component with a proportion of 16.54%, followed by arabinose (14.20%), galactose (13.46%), glucose (13.96%), fucose (13.30%), and rhamnose (10.95%). The contents of fructose and glucuronic acid were 6.73% and 6.97%, respectively, while galacturonic acid showed the lowest proportion at 3.90%. These results confirmed that the extracted product is a typical heteropolysaccharide predominantly composed of neutral pentoses (xylose, arabinose) and hexoses, accompanied by a low content of uronic acids, which indicates the crude polysaccharide has weak acidity and complex branched structural characteristics. These superior properties endow it with great application potential in functional food, natural cosmetics, green agricultural preservation, and biomedical auxiliary materials [25], forming a high‐value comprehensive utilization mode of simultaneous harvesting of essential oil and polysaccharide from *M. alternifolia* biomass.

### 3.5 Techno‐Economic Assessment of the 30 L Scale Integrated Extraction Process

An evaluation of the techno‐economic feasibility of the 30 L scaled batch process was conducted to ascertain the rough production expenses of extracting bioactive compounds from *M. alternifolia*. Since labor expenses, and were highly dependent on the location selected for constructing the pilot plant, Table 5 only outlined the expenditures related to the material costs employed and the energy costs for the involved process stages. According to Table 5, the integrated process for simultaneously extracting 11.5 g of TTO and 55.7 g of polysaccharide from *M. alternifolia* biomass was estimated to incur a total direct material and electricity cost of 176.6 RMB. It was found that *t*‐butanol expenses constituted 65.6% ($\frac{116}{176.6}\times 100\%$) of the overall production costs for the targeted TTO and polysaccharides (Table 5). Therefore, it is meaningful to devise an effective method for the concurrent extraction of TTO and polysaccharides from *M. alternifolia* biomass, which minimizes the utilization of *t*‐butanol. In this study, utilizing the established TPP process for a single extraction under optimized conditions, the material and electricity cost amounted to 15.4 RMB/g of TTO (calculated as 176.6 RMB ÷ 11.5 g), while simultaneously yielding 55.7 g of polysaccharides.

**TABLE 5:** *M. alternifolia*‐derived TTO and polysaccharide extraction cost considering chemicals (A) and energy expenditures in each process step (B) for the 30 L pilot‐scale treatment.

Materials and process step	Quantity	Price reagent	Cost (RMB)
(A) Materials			
Ammonium sulfate	1.8 kg	14 RMB/kg	25.2
*t*‐butanol	14.5 L	20 RMB/L	116
*M. alternifolia* biomass	1 kg	20 RMB/kg	20
Deionized water	10 L	1 RMB/L	10
(B) Process step	Time	Energy (kW/h)	
TPP extraction (motor, 120 W) for (1 kg dry biomass)	100 min	0.2	0.12^a^
Temperature control (1000 W)	2 h	2	1.2^a^
membrane concentration filtration (1.5 kW)	30 min	0.9	0.54^a^
Molecular distillation (1180 W)	5 h	5.9	3.54^a^
Total cost (RMB)			176.6
Extracted TTO quantity^b^			11.5 g
Extracted polysaccharide quantity^b^			55.7 g

^a^ The cost for each process step = Energy × Electricity price (0.6 RMB/(kW·h) used in Fuzhou city.

^b^ A single extraction cycle was chosen to assess the amounts of TTO and polysaccharide extracted.

**FIGURE 4:**
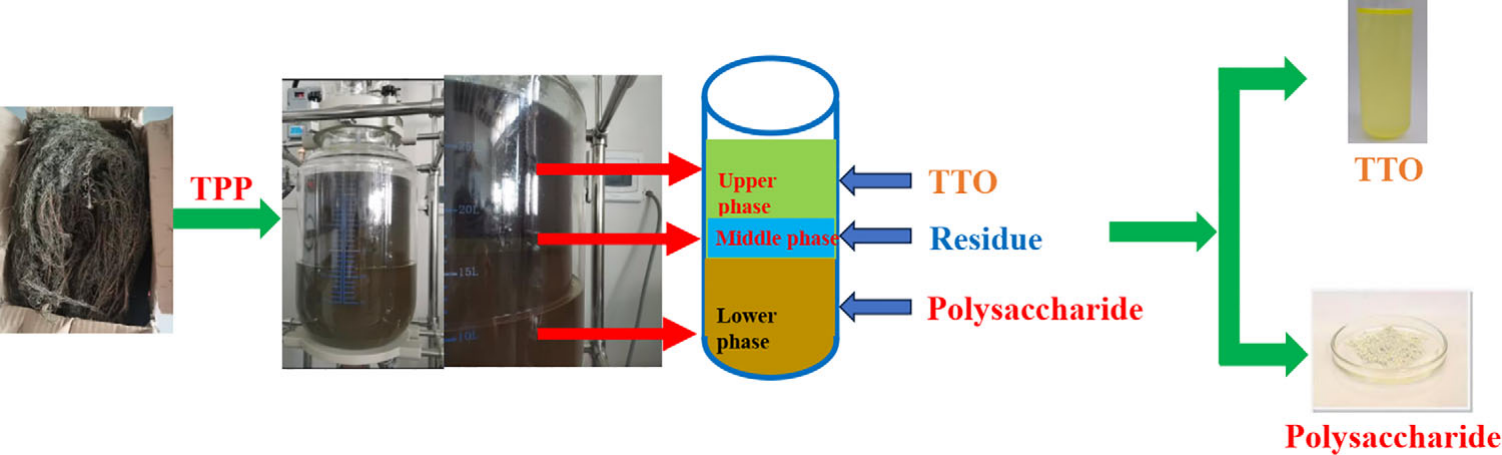
Schematic diagram of the three‐phase partitioning process subjected to integrated extraction TTO and polysaccharide.

In this study, the *t*‐butanol‐mediated TPP strategy showed promise as a potentially sustainable extraction approach. Conventional steam distillation operates at high temperature for 4–6 h to obtain TTO from *M. alternifolia* with a typical yield of 1.4%–1.8% [26], while polysaccharide‐rich residues are completely discarded as waste without recovery. Under mild conditions (60°C), the optimized TPP completes processing within 100 min, delivering a comparable TTO yield of 1.37% while co‐extracting 5.57% polysaccharides. Independent hot‐water polysaccharide extraction requires extra heating and solvent consumption, leading to lower overall efficiency relative to this one‐pot TPP method. In addition, steam distillation consumes large amounts of water and energy, whereas recyclable tert‐butanol in TPP helps cut material costs [27], favouring resource utilization. It should be noted that our study was only carried out at 30 L pilot scale, so full industrial economic evaluation cannot be achieved using present data. To improve the economic competitiveness of the TPP integrated extraction route, future works will focus on optimizing *t*‐butanol distillation recovery efficiency and establishing a full industrial cost model covering all indirect and waste treatment expenditures for comprehensive life‐cycle economic assessment. Nevertheless, TPP acts as a promising green, cost‐efficient process owing to its mild temperature, recoverable solvent and ability to co‐produce high‐value polysaccharides with TTO. To the best of our knowledge, this work was the first to establish a TPP process assisted by (NH_4_)_2_SO_4_ and *t*‐butanol for the efficient extraction and separation of TTO and polysaccharides from *M. alternifolia* (Figure 4). Therefore, TPP serves as an attractive single‐step technology for efficient recovery of both TTO and polysaccharides from *M. alternifolia* biomass.

## 4 Conclusions

This study established an optimized three‐phase partitioning (TPP) process for the simultaneous extraction of tea tree oil (TTO) and polysaccharides from *M. alternifolia*. The optimized TPP method, using (NH_4_)_2_SO_4_ and *t*‐butanol, achieved 1.15% TTO and 5.57% polysaccharide yields in a 30 L reactor. GC–MS comparison between TPP‐extracted oil, steam‐distilled oil and GB/T 26514‐2011 standard revealed that terpinen‐4‐ol (33.19%), the core bioactive marker of TTO, met the national standard range of 30%–48%. Nevertheless, many minor monoterpene components, including *γ*‐terpinene and *p*‐cymene were detected at concentrations below the standard lower limits, which was attributed to the selective partition behavior of the TPP system rather than thermal deterioration of volatile substances. The IC analysis confirmed the recovered polysaccharide was a complex heteropolysaccharide composed of nine monosaccharides dominated by neutral pentoses (xylose and arabinose). Techno‐economic assessment verified the economic feasibility of this integrated process for simultaneous harvesting of two high‐value bioactive products from single biomass feedstock. Future works will center on three core directions: (1) optimizing *t*‐butanol recovery circulation to reduce material costs; (2) refining separation conditions to balance extraction yield and product purity; and (3) conducting systematic antibacterial and antioxidant tests to verify the functional performance of TTO and polysaccharide products.

## 5 Experimental Section

### 5.1 Plant Material and TTO Extraction

The leaves and terminal branches of *M. alternifolia* were purchased from Quanzhou Qiyuan Garden Virescence Co., Ltd., located in Fujian, China. Species identification was performed by Dr. Yangmin Wen. Before the extraction process, these materials underwent air‐drying at ambient room temperature for a duration of at least two weeks. Following, the desiccated material was cut into smaller fragments and subsequently pulverized into a fine powder utilizing a mechanical grinder. The authenticated representative powder samples (No. MA‐20240701) are retained in our laboratory.

The extraction of TTO was accomplished through a TPP separation system. The TPP separation system consists of a suspension prepared from the raw material and water, (NH_4_)_2_SO_4_) (analytical grade, Xilong Scientific Co., Ltd., Shantou, China), and *t*‐butanol (analytical grade, Sinopharm Chemical Reagent Co., Ltd., Beijing, China), with the total volume set at 100 mL in a small‐scale laboratory experiment. After preparing a mixture of the raw material (solid‐liquid ratio), water, (NH_4_)_2_SO_4_, and t‐butanol in specific proportions, it was placed under appropriate temperature for a defined extraction duration (extraction time). During the extraction process, a magnetic stirrer was employed to ensure thoroughly mixing (stirring speed).

### 5.2 Screening of Key Factors in TTO Extraction by TPP Partitioning

The Plackett–Burman Design serves as an effective screening approach for pinpointing key factors from a vast array of elements that impact a given process [28]. A Plackett–Burman Design was employed to investigate the impact of seven factors in a TPP system: the ratio of raw material/water (g/mL), (NH_4_)_2_SO_4_/water (g/mL), the ratio of *t*‐butanol/water (v/v), extraction time (h), extraction temperature (°C), pH, and stirring speed (rpm). Each factor was tested at two levels (high and low), as shown in Table 1. The extraction yield of TTO was used as the response variable to screen and identify the most critical factors influencing the TTO extraction yield in the TPP extraction system.

### 5.3 Optimization of TPP Conditions by Response Surface Methodology (RSM)

On the basis of Plackett–Burman Design results, Box–Behnken Design in RSM was used to design the experiment by Design‐Expert 8.0.6 (Stat‐Ease Inc., Minneapolis, USA), and the numerical optimization was performed to optimize extraction conditions and to obtain the maximum response values [29]. Three factors, namely, (NH_4_)_2_SO_4_/water (X_1_), *t*‐butanol/water (X_2_), and extraction time (X_3_), were the independent (input) variables. Independent variables with three variation levels were as follows: X_1_ (NH_4_)_2_SO_4_/water: 0.1, 0.2, and 0.3 g/mL), X_2_ t‐butanol/water: 0.5:1, 1.5:1, and 2.5:1 mL/mL), X_3_ (extraction time: 30, 90, and 150 min), as shown in Table 6. A total of 17 experiments with three center points based on Box–Behnken experimental design were performed, and the results are presented in Table 6. The second‐order polynomial equation [30] reflecting the relationship between the response values and independent variables was expressed as Equation (2):

(2) $$Y=A_{0}+\sum_{i=1}^{3}A_{i}X_{i}+\sum_{i=1}^{3}A_{\mathrm{ii}}{X_{i}}^{2}+\sum_{i=1}^{3}+\sum_{j\;=1}^{3}A_{i,j}X_{i}X_{J}$$

**TABLE 6:** Box–Behnken experimental design and the results for yield of TTO.

Run numbers	Coded variable levels			Extraction efficiency (%)
	X_1_: (NH_4_)_2_SO_4_/water	X_2_: *t*‐butanol/water	Time	
1	1	1	0	1.27
2	0	0	0	1.34
3	0	1	1	1.11
4	1	0	−1	1.06
5	1	−1	0	1.17
6	−1	0	1	1.01
7	0	0	0	1.41
8	−1	−1	0	0.82
9	0	1	−1	1.13
10	−1	0	−1	1.02
11	0	0	0	1.32
12	−1	1	0	0.98
13	0	0	0	1.38
14	1	0	1	1.21
15	0	−1	−1	1.06
16	0	0	0	1.39
17	0	−1	1	1.06

where, Y is the response value (yield of TTO, %), and A_0_, A_i_, A_ii_, and A_ij_ (i≠j) are the coefficients of variables for the intercept, linear, quadratic, and interactive terms, respectively.

### 5.4 Extraction of TTO and Polysaccharide Using TPP in 30 L Scale

For the 30 L pilot experiment, all solid‐liquid and solvent ratios matched the optimized 100 mL conditions with only the total volume linearly scaled up and all raw materials proportionally increased. Briefly, the 1 kg sample of *M. alternifolia* powder was dissolved with 10 L water, then 1.8 kg of (NH_4_)_2_SO_4_ was added to the crude extract prepared and vortexed gently, followed by addition of 14.5 L *t*‐butanol. The pH of the crude extract was kept at natural condition. The mixture was gently vortexed at a stirring speed of 50 rpm and then extracted for 100 min at 60°C controlled by a water bath (1000 W).

### 5.5 TTO and Polysaccharide Yield Determination

After extraction, the mixture was transferred to a separatory funnel and settled to complete phase separation. The upper *t*‐butanol‐rich organic phase containing TTO was collected with a Pasteur pipette, then concentrated under reduced pressure on an RE52CS‐1 rotary evaporator (Shanghai Yarong Biochemical Instrument Co., Ltd., Shanghai, China) at 50°C for 5 min to strip off t‐butanol. The resulting crude TTO was dried over anhydrous Na_2_SO_4_ and filtered to remove desiccant residual. GC‐MS analysis (Agilent 5975C, Agilent Technologies, Santa Clara, USA) verified that no *t*‐butanol residue was detectable in the final TTO product. Then the TTO was precisely weighed by optically analytical balance. The extraction yield of the crude oil was determined by the Equation (3).

(3) $$\mathrm{TTO}\;\mathrm{yield}\;\left(\%\right)=\frac{\mathrm{Mss}\;\mathrm{of}\;\mathrm{oil}\;\left(\mathrm{mg}\right)}{\mathrm{Mass}\;\mathrm{of}\;\mathrm{leaves}\;\mathrm{powder}\;\left(\mathrm{mg}\right)}\;\times 100\%$$

The (NH_4_)_2_SO_4_‐rich lower phase was purified for polysaccharides. The aqueous solution was dialyzed with 8–12 kDa bags against deionized water at 25°C for 48 h to remove salts and small impurities. Four volumes of pre‐cooled 95% ethanol were added under stirring (100 rpm), and the mixture was kept at 4°C for 12 h for full polysaccharide precipitation. The precipitate was collected by centrifugation at 6000 rpm (4°C, 15 min) and washed three times with absolute ethanol. After redissolution in ultrapure water, the sample was frozen at −40°C for 6 h and freeze‐dried under 0.08 mbar vacuum for 48 h to obtain purified polysaccharide powder for subsequent analysis. The polysaccharide content was determined via the phenol–sulfuric acid method [31]. The polysaccharide extraction yield was calculated using Equation (4).

(4) $$\mathrm{Polysaccharide}\;\mathrm{yield}\;\left(\%\right)=\frac{\mathrm{Mss}\;\mathrm{of}\;\mathrm{polysaccharide}\;\left(\mathrm{mg}\right)}{\mathrm{Mass}\;\mathrm{of}\;\mathrm{leaves}\;\mathrm{powder}\;\left(\mathrm{mg}\right)}\;\times 100\%$$

### 5.6 The Compound Analysis of TTO and Polysaccharides

The compounds of the extracted TTO were analyzed by a gas chromatography‐mass spectrometry [32]. The Agilent 5975C detector, equipped with a PC‐Wax column (30 m long, 250 µm wide, 0.25 µm internal diameter, at 450°C), was employed. The oven program remained active, with an equilibrium time of 0 min, and operated for 36 min. The heater was set at a temperature of 240°C. Helium, flowing at a rate of 0.7 mL/min, acted as the carrier gas. Mass spectrometry was performed in EMV mode at a relative voltage of 59 eV, scanning from 50 to 550 amu. The mass spectrometer was equipped with a gold—standard quadrupole analyzer operating at 150°C (maximum 200°C). The temperature of the MS source was 240°C (maximum 250°C).

The hydrolyzed polysaccharide samples were analyzed by ion chromatography ((ICS‐5000+, Thermo–Fisher Scientific, Waltham, USA)) equipped with a pulsed amperometric detector for qualitative identification and quantitative calculation of each monosaccharide component.

### 5.7 Statistical Analysis

The trials of PB and RSM were designed and data analysis by Design‐Expert 8.0.6 (Stat‐Ease, Inc., Minneapolis, USA). The rest trials were carried out in triplicate (*n* = 3), and data were performed by MicroCal Origin 8.5 software (OriginLab Corporation, Northampton, USA) and presented as means ± standard deviation.

## Author Contributions

**Huan Liao**: conceptualization, formal analysis, methodology, investigation, writing. **Simin Wang**: conceptualization, formal analysis, methodology, investigation. **Ying Wu**: investigation, formal analysis. **Zhihong Chen**: writing – review and editing. **Wentao Chen**: methodology, formal analysis. **Leiwen Xiang**: supervision, editing. **Yangmin Wen**: conceptualization, formal analysis, methodology, investigation, writing. **Youcai Zhou**: conceptualization, writing – original draft, funding acquisition.

## Conflicts of Interest

The authors declare no conflicts of interest.

## Supporting information




**Supporting File 1**: cbdv71748‐sup‐0001‐SuppMat.docx

## Data Availability

The data that support the findings of this study are available from the corresponding author upon reasonable request.
