# Supplementary material for: Integrated Extraction of Tea Tree Oil and Polysaccharides From Melaleuca alternifolia Using Three‐Phase Partitioning: Process Optimization, Techno‐Economic Analysis, and Sustainability Evaluation
Source: Chem Biodivers. 2026 Sep 25;23(9):e71748. doi: 10.1002/cbdv.71748 (PMC13614291; doi:10.1002/cbdv.71748)
Supplement: Supplementary file 1 [file CBDV-23-e71748-s001.docx]

Supplementary Material

Supporting information for this article is available on the WWW under http://dx.doi.org/10.1002/MS-number.

**Table S1** The design and corresponding results

| No. | Factors and levels | | | | | | | | extraction efficiency (%) |
| --- | --- | --- | --- | --- | --- | --- | --- | --- | --- |
|  | The ratio of raw/water  (g/mL) | (NH_4_)_2_SO_4_/water (g/mL) | t-butanol  /water (v/v) | Time (min) | Temperature  (℃) | pH | Stirring speed (r/min) | Error item |  |
| **1** | 1:10 | 0.1 | 2.5:1 | 30 | 60 | 5 | 250 | 1 | 1.29 |
| **2** | 1:30 | 0.3 | 0.5:1 | 150 | 60 | 5 | 50 | 1 | 1.35 |
| **3** | 1:30 | 0.3 | 2.5:1 | 30 | 80 | 5 | 50 | -1 | 1.05 |
| **4** | 1:10 | 0.1 | 2.5:1 | 150 | 60 | 9 | 50 | -1 | 1.21 |
| **5** | 1:10 | 0.3 | 0.5:1 | 150 | 80 | 5 | 250 | -1 | 1.31 |
| **6** | 1:10 | 0.3 | 2.5:1 | 30 | 80 | 9 | 50 | 1 | 1.47 |
| **7** | 1:30 | 0.3 | 2.5:1 | 150 | 60 | 9 | 250 | -1 | 1.04 |
| **8** | 1:30 | 0.3 | 2.5:1 | 150 | 80 | 5 | 250 | 1 | 1.03 |
| **9** | 1:30 | 0.1 | 0.5:1 | 150 | 80 | 9 | 50 | 1 | 0.45 |
| **10** | 1:10 | 0.1 | 0.5:1 | 30 | 80 | 9 | 250 | -1 | 0.96 |
| **11** | 1:30 | 0.3 | 0.5:1 | 30 | 60 | 9 | 250 | 1 | 0.74 |
| **12** | 1:30 | 0.1 | 0.5:1 | 30 | 60 | 5 | 50 | 1 | 0.44 |

**Table S2** The components of TTO derived by TTP method

| No. | Retention time | Components | CAS# | proportion |
| --- | --- | --- | --- | --- |
| 1 | 6.37 | α - Pinene | 7785-70-8 | 1.090% |
| 2 | 7.384 | Camphene | 79-92-5 | 0.347% |
| 3 | 9.007 | β - Pinene | 127-91-3 | 30.700% |
| 4 | 9.477 | α-terpinene | 99-86-5 | 14.957% |
| 5 | 10.759 | β-myrcene | 123-35-3 | 0.478% |
| 6 | 11.369 | 1,8-cineole | 470-82-6 | 6.167% |
| 7 | 12.638 | γ-Terpinene | 99-85-4 | 3.727% |
| 8 | 13.571 | Terpinolene | 586-63-0 | 0.327% |
| 9 | 15.143 | Allocimene | 7216-56-0 | 0.015% |
| 10 | 16.599 | Linalool oxide | 5989-33-3 | 0.008% |
| 11 | 17.063 | p-Cymene | 99-87-6 | 0.019% |
| 12 | 17.937 | (Z)-β-Terpineol | 7299-40-3 | 0.012% |
| 13 | 19.079 | （E）-p-Menth-2-en-1-ol | 29803-81-4 | 0.024% |
| 14 | 19.699 | 1-Terpineol | 586-82-3 | 0.153% |
| 15 | 20.908 | Terpinen-4-ol | 562-74-3 | 33.193% |
| 16 | 21.563 | α-Cedrene | 3853-83-6 | 0.463% |
| 17 | 21.654 | β-Terpineol | 138-87-4 | 0.207% |
| 18 | 22.142 | (+)-Valencene | 489-39-4 | 0.233% |
| 19 | 22.764 | α-Terpineol | 98-55-5 | 5.225% |
| 20 | 23.191 | Nootkatone | / | 1.072% |
| 21 | 24.189 | Aromadendrene | 25246-27-9 | 0.176% |
| 22 | 25.215 | (+)-Ledene | 21747-46-6 | 0.196% |
| 23 | 25.751 | Calamenene | 17334-55-3 | 0.071% |
| 24 | 26.426 | Δ-Cadinene | 483-76-1 | 0.173% |
| 25 | 26.839 | Cadina-1,4-diene | 16728-99-7 | 0.061% |
| 26 | 27.642 | Dehydrocalamenene | 483-77-2 | 0.031% |
| 27 | 29.803 | Methyleugenol | 93-15-2 | 0.193% |
| 28 | 31.003 | Terpinen-4-ol | 552-02-3 | 0.163% |
| 29 | 31.277 | (-)-Epieudesmol | 1000150-05-1 | 0.270% |
| 30 | 31.826 | (+) -Terpinen-4-ol | 51371-47-2 | 0.124% |
| 31 | 32.01 | β-Eudesmol | 473-15-4 | 0.125% |
| Total |  |  |  | 100% |
